# Associations Between Physical Activity in Pregnancy and Maternal, Perinatal, and Neonatal Parameters: A Single-Center Prospective Cohort Study

**DOI:** 10.3390/jcm14072325

**Published:** 2025-03-28

**Authors:** Paulina Majewska, Anna Szablewska

**Affiliations:** Department of Obstetric and Gynaecological Nursing, Institute of Nursing and Midwifery, Faculty of Health Sciences with the Institute of Maritime and Tropical Medicine, Medical University of Gdansk, 80-210 Gdansk, Poland; paulina.budna.00@gumed.edu.pl

**Keywords:** maternal physical activity, pregnancy, neonatal

## Abstract

**Background**: Physical activity during pregnancy plays an important role in influencing the course of pregnancy, the health of the mother, and neonatal outcomes. Regular exercise can positively affect maternal well-being, reduce the risk of pregnancy-related complications, and support optimal fetal development. Additionally, physical activity may contribute to a reduced need for C-sections and better postpartum recovery. Despite these benefits, global trends indicate a decline in physical activity levels, exacerbated by lifestyle changes such as remote work. This highlights the importance of promoting healthy habits among women of reproductive age to improve perinatal outcomes and the long-term health of both mothers and their children. **Objective:** The aim of this study was to investigate the effects of physical activity during pregnancy on the health of mothers and babies after birth. **Methods**: A prospective cohort study was conducted in a tertiary care hospital in northern Poland from October 2024 to December 2024. Participants were 205 pregnant women with no medical contraindications to physical activity. The group was selected on the basis of a questionnaire with original questions and the Get Active Questionnaire for Pregnancy (GAQ-P). The effects of physical activity during pregnancy on maternal and infant health after delivery were assessed using questionnaire data and medical records. Frequency analysis supported by chi-squared coefficient; Cramer’s V coefficient and Spearman’s rank correlation were used to answer the research questions. **Results**: The study showed that the frequency of physical activity had an effect on the incidence of perineal trauma, the baby’s birth weight, and the baby’s degree of saturation after birth. The intensity of physical activity during pregnancy may influence the duration of the first stage of labor, while the duration of physical activity may influence the duration of the second stage of labor and reduce the number of operative deliveries. **Conclusions**: Although physical activity has a huge impact on the course of pregnancy and the health of both mother and child after birth, more research is needed to draw clear conclusions. In our study, a beneficial effect of physical activity on the reduction in cesarean sections can be observed. However, the effects on perineal injuries, length of labor, and birth weight require further research, as our findings indicate that higher exercise frequency was associated with both positive outcomes, such as fewer cesarean sections, and potential risks, including an increase in macrosomia and perineal tears. A broader analysis of co-factors influencing these results is needed to fully understand these relationships.

## 1. Introduction

Physical activity (PA) is one factor that benefits health regardless of age [1]. The World Health Organization (WHO) recommendations are for physical activity of moderate intensity for at least 150 min per week. Studies show that approximately 28% of adults aged ≥ 18 years and 81% of adolescents aged 11–17 years do not meet these recommendations [2]. While the WHO estimates provide a global perspective, physical inactivity levels can vary significantly between countries, with national studies often reporting higher rates of inactivity, particularly in Europe and other specific regions [3]. Low levels of physical activity in all age groups may, therefore, affect the future health of the general population, increasing the risk of various diseases. The American College of Obstetricians and Gynecologists (ACOG) takes a similar position regarding physical activity recommendations for pregnant women [2,4]. Physical activity is extremely important for individuals of reproductive age, including those who identify as women. Currently, there is a worldwide trend towards a sedentary lifestyle. Remote work and limited physical activity can lead to difficulties in becoming pregnant. Public awareness of how to prepare women’s and men’s bodies for pregnancy and consequently ‘program’ the health of the future child is growing, but it needs to be further developed and disseminated [4].

Physical activity has many present and future benefits for both mother and child. In women, the literature describes a reduced risk of pre-eclampsia, pregnancy-induced hypertension, preterm births, and intrauterine deaths [5]. Many studies confirm reduced weight gain in women and a reduced risk of gestational diabetes and cesarian section [4,5,6,7]. Women who exercise during pregnancy have a better mood, higher self-esteem, less anxiety, and a lower risk of depression [8]. The mother’s moderate physical activity has a positive effect on the child’s neurodevelopment and can protect against possible metabolic diseases. It is also an element in the development of a healthy immune system [1,9].

A study of pregnant women’s knowledge of the effects of physical activity on pregnancy, childbirth, and the health of the mother and child showed that a significant proportion of women actually know about the positive effects, but it is worrying that in many cases, in addition to family and friends, some doctors advise against physical activity during pregnancy [10]. The Internet is the main source of information for patients [10]. Despite the availability of a list of contraindications to physical activity in pregnancy and tools to assess them, women are often misclassified and discouraged from physical activity more than they should be.

Despite growing patient awareness and numerous studies on the effects of physical activity on pregnancy, there are still many inaccuracies in the literature. Unfortunately, the level of physical activity in Poland is lower than recommended, so we undertook the following study to fill a gap in knowledge and understanding of the effects of physical activity on women and newborns [11].

The aim of this study was to investigate the relationship between physical activity during pregnancy and selected maternal parameters, the course of labor, and specific neonatal outcomes.

## 2. Materials and Methods

### 2.1. Study Design

The study presented here was a prospective, single-center study involving a group of 205 pregnant women (ethnic minorities were under-represented in the study group given that in Poland, ethnic minorities make up a relatively small proportion of the total population, so we did not distinguish between ethnic groups). The study followed a two-stage design. We used a questionnaire consisting of self-reported questions in the form of a medical interview and Get Active Questionnaire for Pregnancy. In the second stage, we analyzed electronic medical records. We followed the STROBE guidelines [12]. All procedures were performed in accordance with the tenets of the World Medical Association (WMA) Declaration of Helsinki for research involving human subjects and approved by the Bioethics Committee of the Medical University of Gdansk, No. NKBBN/406-1/2024.

### 2.2. Setting

The study was conducted in a tertiary (level III) referral hospital in northern Poland (Polish hospitals are classified into 3 levels: I—care of pregnancy, delivery, physiological puerperium and healthy newborns (possibly with minor disorders), II—pregnancies with moderate complications, and III—high-risk pregnancies). Data were collected from October 2024 to December 2024.

### 2.3. Participants

Participants in the study were women in the antenatal clinic of a tertiary referral hospital. Participants were recruited at the beginning of the third trimester of pregnancy (after the 28th week of gestation). This timing allowed for a thorough evaluation of the obstetric clinical situation, including potential contraindications to physical activity, especially concerning cervical shortening and the placental condition and location. Additionally, recruiting women at this stage enabled us to assess their physical activity levels throughout the entire pregnancy, while also providing the opportunity to plan for physical activity for the remainder of the pregnancy through the questionnaire.

Each woman was informed that she was volunteering for the study, and each gave written consent for access to her and her baby’s medical records if she gave birth at that hospital. The principal investigator interviewed each pregnant woman and allowed her to ask any questions.

Two hundred thirty women who met the inclusion criteria were invited to take part in the study. In the end, 213 pregnant women who returned questionnaires to us took part. After excluding 8 pregnancies that clinically could have represented an error in the interpretation of the results (high-risk pregnancies that could end in preterm delivery, affecting the health of the newborn), 205 pregnant women were included in the study.

**Figure 1:**
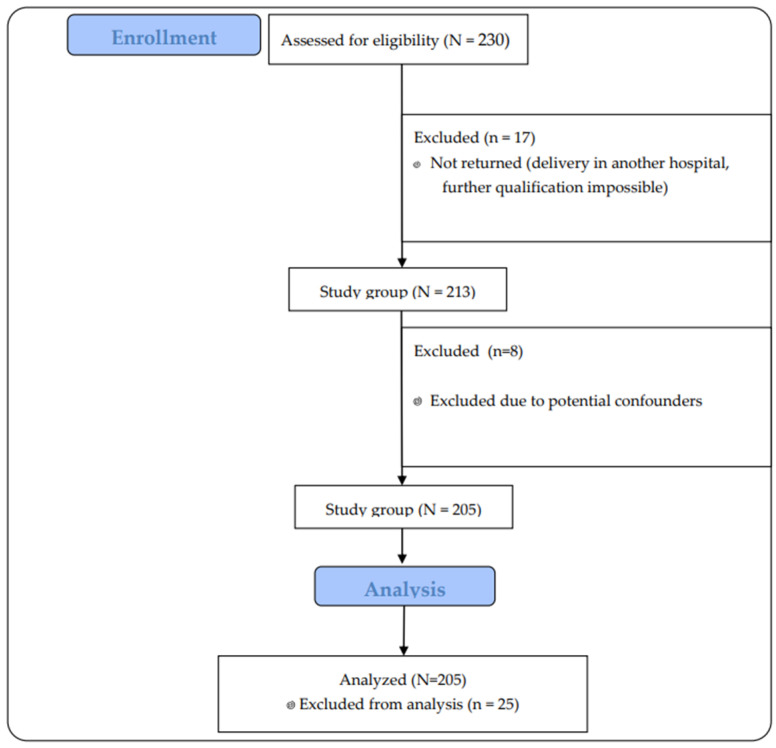
Flow of participants through the study.

The participants’ flow through the study is presented in Figure 1.

### 2.4. Inclusion and Exclusion Criteria

Inclusion criteria were pregnant women over 18 years of age, understanding Polish, attending an antenatal clinic, and giving birth in the same hospital. We excluded multiple pregnancies and pregnant women with an anterior placenta from the study, as these pregnancies have a significant risk of preterm delivery and low neonatal birth weight, which could be confounding factors in the interpretation of the results. Additionally, we considered all contraindications to physical activity listed in the GAQ-P questionnaire, but the rest of the recruited participants did not have any other contraindications mentioned in the questionnaire.

### 2.5. Data Collection Tools

The questionnaire is divided into two parts—the first is a self-administered questionnaire on socio-demographic factors, the course of the current pregnancy, previous obstetric history, and antenatal care. In the second part, the patients answered questions from the Polish version of the Get Active Questionnaire for Pregnancy (GAQ-P) [13]. This is a tool about the woman’s current state of health, which made it possible to exclude women with contraindications to physical activity. The questionnaire included questions about systemic diseases, e.g., respiratory, cardiovascular, epilepsy, diabetes, thyroid disease, eating disorders, anemia, and hypertension. Patients also answered questions about the course of their current pregnancy, i.e., whether they had any conditions such as intrauterine growth restriction, small for gestational age, genital bleeding, preterm premature rupture of membranes, carotid insufficiency, or circular suture. Patients were asked about their obstetric history—recurrent miscarriages and preterm births. By patients answering YES or NO to the above questions, we were able to identify a group of pregnant women who had contraindications to physical activity or who themselves were afraid of physical activity due to a severe obstetric history. The next task for the women was to determine their previous physical activity at 3 stages:

- 6 months before pregnancy;
- up to the time of completing the questionnaire;
- the expected target at the end of the pregnancy.

The frequency, intensity, duration, and type of physical activities most commonly chosen by pregnant women were also reported.

### 2.6. Bias

To make the study more reliable, we checked the information on the questionnaires against the patients’ electronic medical records. This allowed us to minimize potential errors due to participant recall or subjective assessment.

Participants with twin pregnancies and placenta previa were excluded from the study. Our decision was based on the clinical situation of the patients, as these conditions predispose to bleeding and preterm birth, and women in the more favorable situation are often advised to limit physical activity for therapeutic reasons. Including these patients in the study could have biased the results in the group of women without these burdens. This made the group more homogeneous and the results more reliable.

### 2.7. Variables and Outcome Measures

A correlation model was chosen for the study, so we refer to all variables as co-occurring.

Maternal variables.

The first variable we examined in relation to the impact of physical activity was perineal injury in postpartum women. The literature recommends using the Sultan classification to classify perineal injuries according to the depth of the injury [14,15]. Depending on the depth and the muscles damaged, we distinguish the following perineal injuries:

- Lack of (when there is no visible damage);
- 1st (only a break in the continuity of the skin);
- 2nd (damage to the perineal muscles, without injury to the anal sphincter);
- 3rd, which we divide into 3a, 3b, and 3c (3a—less than 50% of the thickness of the external anal sphincter is damaged; 3b—more than 50% of the thickness of the external anal sphincter is damaged; 3c—both the external and internal anal sphincters are damaged);
- 4th (the anal sphincter and the anal mucosa are damaged).

Studies show that about 85% of women experience perineal trauma during vaginal birth. The most common injury is a second-degree tear, depending on the source [14]. According to Baryon [16], an episiotomy is comparable to a second-degree perineal tear, so patients with an episiotomy (n = 3) were included in the group of patients with a second-degree injury.

The second factor analyzed in relation to physical activity was the duration of labor for vaginal births. This variable was not analyzed in the case of cesarian section and attempted vaginal births that ended in cesarian section. In Poland, the document that defines the time frame for the different stages of labor is the Standard of Perinatal Care [17].

- The first stage of labor is recognized when there are regular contractions leading to progression of labor, i.e., dilation of the cervix and descent of the baby into the birth canal. Progress is indicated when dilation is slower than 0.5 cm per hour.
- The second stage of labor begins when the cervix is fully dilated (10 cm) and the baby is born. It can last up to 2 h, or 3 h if an epidural is used.
- The third stage of labor lasts from the birth of the baby to the delivery of the placenta. The maximum duration is 30 min.

Another factor influencing how the relationship was rated was how the pregnancy ended. We have distinguished 2 types of birth:

- Vaginal birth (VB)
- Cesarian section (C-section)

Due to the fact that operative delivery can also be a vaginal delivery and the small size of this group (n = 7), patients who gave birth with the aid of a vacuum were included in the vaginal delivery group.

To assess the influence of physical activity in pregnant women, the effect on maternal hemoglobin levels was tested. Moderate physical activity has been shown to influence hemoglobin levels, with some studies reporting a potential increase [18], while others highlight the role of increased metabolic demands and oxidative stress, which may contribute to anemia, particularly in conditions of inadequate energy and nutrient intake [19]

**Figure 2:**
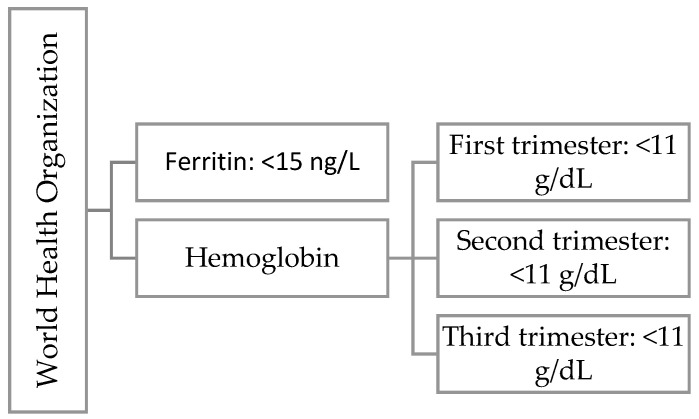
WHO definition for iron deficiency during first trimester of pregnancy.

**Figure 3:**
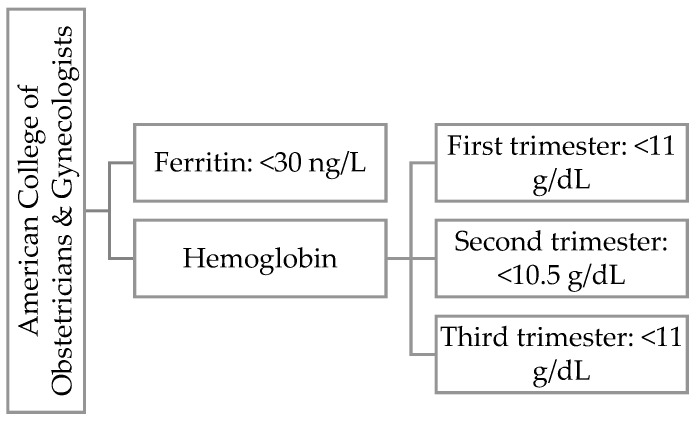
ACOG definition for iron deficiency during any trimester of pregnancy.

Hemoglobin is a protein found in red blood cells that transports oxygen from the lungs to other organs and tissues. In pregnant women, we observe the phenomenon of physiological anemia caused by an increase in plasma volume. Depending on the literature, there are differences in the classification of anemia [20,21,22]. In Figure 2 we present WHO definition for iron deficiency during first trimester of pregnancy and Figure 3 ACOG definition for iron deficiency during any trimester of pregnancy.

Neonatal variables.

Another element we looked at was the effect of physical activity on the baby’s birth weight. In the literature, the breakdown of neonates by birth weight is as follows [23,24]:

- Extremely low birth weight, ELBW: <1000 g;
- Very low birth weight, VLBW: 1000–1499 g;
- Low birth weight, LBW: 1500–2499 g;
- Normal birth weight, appropriate: 2500–3999 g;
- Macrosomia: >4000 g.

We examined the association between physical activity and selected measures of postnatal infant assessment: saturation, APGAR scale, and umbilical arterial blood pH.

On the first day of life, pulse oximetry screening is performed on the infant’s right lower limb, which allows rapid detection of asymptomatic conduction-dependent heart defects. The normal range of blood oxygen saturation in Poland is considered to be 95–100% [17].

The APGAR score allows a quick assessment of the baby’s condition after birth without interfering with the infant’s care. We gave 0 to 2 points in 5 categories:

- fetal heart rate;
- respiration;
- response to stimuli;
- muscular tension;
- skin color.

Assessments are made at 1 and 5 min of the baby’s life [25]. However, the most accurate and objective assessment is to combine the APGAR saccade score with analysis of umbilical-cord blood gas analysis. The normal blood pH range is 7.30–7.20, while a value below 7.20 may indicate metabolic acidosis and hypoxia in the newborn [25,26].

We also looked at the relationship between the type of physical activity, the age of the pregnant woman, fertility, and the amount of physical activity.

### 2.8. Statistical Analysis

Results were compiled using Statistica 13.3. An alpha level of 0.05 was chosen for the overall study, frequency analysis was supported by chi-square, and Cramer’s V coefficient was used to answer the research questions posed. Correlation analysis for ordinal and quantitative variables was enriched by evaluating the Spearman rank correlation coefficient.

## 3. Results

### 3.1. Characteristics of the Study Group

A total of 230 women were enrolled in the study, with 25 excluded based on the predefined exclusion criteria. The questionnaires and medical records of 205 women without medical contraindications to physical activity during pregnancy were analyzed. Table 1 presents the characteristics of the participants.

#### 3.1.1. Selected Pregnancy-Related Variables

Table 2 presents the values of selected pregnancy-related variables, including age at delivery, pre-pregnancy BMI, gestational weight gain, birth weight, gestational age at birth, cord blood pH, and maternal blood hemoglobin. One of the study variables was pre-pregnancy BMI, and the results related to this variable are presented in a separate analysis.

#### 3.1.2. Assessing the Impact of Physical Activity on Selected Factors

The first factor assessed in relation to physical activity during pregnancy was the incidence of perineal injuries in women following childbirth. Table 3 presents an analysis of the incidence of perineal injuries postpartum in relation to the levels of physical activity during pregnancy.

The analysis revealed a significant association between the frequency of physical activity during pregnancy and the incidence of perineal injury, χ^2^(9) = 17.96, *p* = 0.036, V = 0.17. The strength of this association was weak. It was observed that the number of women without perineal injury decreased as the frequency of physical activity increased. However, the association between the intensity (χ^2^(6) = 2.34, *p* = 0.886, V = 0.08) and duration of physical activity (χ^2^(9) = 9.30, *p* = 0.410, V = 0.12) with the incidence of episiotomy was not statistically significant. The second factor analyzed for its association with physical activity was the duration of labor. A summary of the association analysis is provided in Table 4.

The analysis revealed a positive correlation between the frequency and intensity of physical activity during pregnancy and the duration of the first stage of labor. The second stage of labor appeared to be significantly correlated with the duration of physical activity. The duration of both the first and second stages of labor showed a weak positive correlation with measures of physical activity during pregnancy. Higher levels of physical activity were associated with longer durations of labor. Another factor analyzed in relation to physical activity was the mode of delivery, which is summarized in the table below [Table 5].

The frequency (χ^2^(3) = 3.94, *p* = 0.268, V = 0.14) and intensity (χ^2^(3) = 2.35, *p* = 0.308, V = 0.11) of physical activity during pregnancy did not show a significant correlation with the mode of birth. However, an association was observed between the mode of birth and the duration of physical activity (χ^2^(3) = 7.67, *p* = 0.053, V = 0.19). The associations between the mode of birth and physical activity during pregnancy were weak. As shown in the table above, the number of operative births decreased as the level of physical activity during pregnancy increased. The effect of physical activity on hemoglobin levels was also assessed. A summary of this analysis is presented in the Table 6.

**Figure 4:**
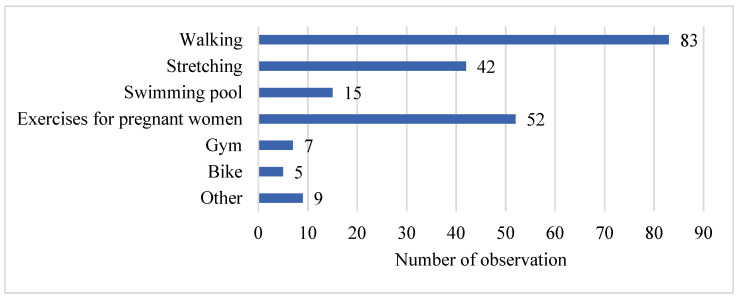
Physical activity during pregnancy among women in their first pregnancy.

However, the results showed no correlation between physical activity and hemoglobin levels. The Spearman’s rank correlation coefficient (*r_s_)* indicated a very weak association. In an additional analysis, the type of physical activity undertaken by the women during their first pregnancy was assessed. A summary of these results is presented in the figure below (Figure 4).

Walking was the most commonly reported type of physical activity among women during their first pregnancy. Other activities, such as exercises for pregnant women, stretching, and swimming in a pool, were also reported but with significantly lower frequency compared to walking. The results demonstrated a significant negative correlation between the number of different physical activities during pregnancy and the parity of the women surveyed (*r_s_* = −0.38, *p* < 0.001), indicating a moderate association. Specifically, the number of different types of physical activity decreased with subsequent pregnancies. Additionally, the level of physical activity was re-evaluated for its association with maternal age. A summary of this analysis is presented in Table 7.

The relationship between maternal age and physical activity during pregnancy was not found to be statistically significant. A similar analysis was conducted to assess the association between physical activity and birth weight, with the results presented in Table 8.

The results indicated that the baby’s birth weight was not significantly correlated with the level of maternal physical activity during pregnancy. Various physical activity parameters were thoroughly evaluated in relation to birth weight outcomes. A detailed summary of these measurements is presented in Table 9.

The frequency of physical activity during pregnancy was found to be significantly associated with the infant’s birth weight, χ^2^(6) = 15.89, *p* = 0.014, V = 0.20. The association was weak. It was observed that as the frequency of physical activity increased, the incidence of low-birth-weight infants also increased, up to a high frequency of 5–7. In this group, the number of infants with normal birth weight decreased, while the incidence of macrosomia increased. Additionally, as the frequency of physical activity during pregnancy increased, the number of infants classified as small for gestational age (SGA) decreased. Furthermore, the analysis revealed that the intensity (χ^2^(4) = 6.42, *p* = 0.170, V = 0.13) and duration (χ^2^(9) = 10.14, *p* = 0.119, V = 0.16) of physical activity during pregnancy were not significantly correlated with birth weight.

The table below [Table 10] summarizes the assessment of the association between physical activity levels and selected infant health measures.

The results showed that neonatal oxygen saturation was significantly correlated with the frequency and intensity of women’s physical activity during pregnancy. The associations were positive but weak. Higher levels of physical activity during pregnancy were associated with higher neonatal oxygen saturation scores. However, Apgar scale scores, measured at both the first and fifth minute, did not show significant correlations with physical activity during pregnancy. Cord blood pH was significantly correlated with the frequency and duration of women’s physical activity during pregnancy. The associations were weak and negative. As physical activity increased, cord blood pH decreased. An additional analysis examined the association between the number of different physical activities performed by women during pregnancy and the baby’s birth weight. This analysis found no significant correlation between birth weight and the number of different physical activities the women engaged in during pregnancy (*r_s_* = 0.05, *p* = 0.447).

## 4. Discussion

Our study suggests that physical activity during pregnancy may positively influence birth outcomes. Although we observed a tendency toward fewer cesarean sections among physically active women, this association was not statistically significant. Furthermore, we identified a significant relationship between the frequency of physical activity and birth weight, with higher levels of activity associated with both a reduced incidence of low birth weight and an increased occurrence of macrosomia. Another notable finding was the positive correlation between physical activity and neonatal oxygen saturation, alongside a weak negative correlation with cord blood pH. The first analysis in our study examined the association between physical activity and perineal injury following childbirth. Our analysis of the relationship between physical activity and perineal injury revealed a complex pattern. While ACOG [4] suggests that physical activity during pregnancy may help prepare the perineum for childbirth and reduce the risk of injury, our results did not demonstrate a clear protective effect. Our results in Table 3 suggests that physically inactive women were more likely to have no perineal tears, whereas an increase in physical activity frequency was associated with a greater incidence of perineal injuries, particularly first- and second-degree tears. Notably, those who engaged in physical activity 3–4 times per week had a higher proportion of first-degree tears, while those who exercised only 1–2 times per week had more second-degree tears. The highest frequency group (5–7 times per week) showed a mixed distribution of injuries, without a consistent protective trend. In a study conducted by Uccella, Manzoni, et al. [27], a lower rate of episiotomy and second-degree perineal lacerations was observed among women who practiced sports specifically targeting the perineal muscles and continued this practice throughout pregnancy. The authors suggested that physical exercise focused on the pelvic floor may increase the elasticity of this area, potentially leading to better outcomes during delivery. However, other studies have found no significant relationship between physical activity during pregnancy and perineal injuries, including episiotomy and lacerations [28]. These findings highlight the need to consider additional factors that may influence perineal injury beyond physical activity alone. For instance, maternal age, tissue elasticity, fetal weight, and obstetric interventions could play a significant role. Additionally, the type, intensity, and duration of physical activity may impact perineal outcomes in ways that were not fully captured in our study. Future research should focus on exploring these potential confounders to determine whether specific forms of exercise contribute to improved perineal resilience or if other factors mediate this relationship.

Another aspect of our study was the effect of physical activity on the duration of labor. Unexpectedly, we observed a positive correlation between physical activity and the length of both the first and second stages of labor. These findings contrast with those of Ribeiro et al. [6] and Barakat et al. [29], who reported that physical activity is associated with a reduction in the total duration of labor, particularly in the first stage. Our results suggest that the duration of labor may be more strongly influenced by other factors, such as parity or maternal physiology, rather than by physical activity alone. A large proportion of our study group consisted of first-time mothers (n = 101), and it is well known that first-time pregnancies are often associated with longer labor and a higher incidence of perineal injuries. However, given the relatively small number of nulliparous women in our sample, the data structure does not allow for a comprehensive analysis of parity-related differences in physical activity effects. Therefore, we propose that future research focusing exclusively on more homogenous cohorts (for example first-time mothers) could provide further insights into this issue and clarify the role of physical activity in labor duration.

Our study demonstrated that the frequency and intensity of physical activity during pregnancy had no significant effect on the type of delivery. However, we observed an association between the duration of physical activity and a reduction in the incidence of cesarian sections. These findings align with the results of DiPietro et al. [30] in their Umbrella Review, where it was shown that women who engaged in regular exercise before and during pregnancy were less likely to undergo a cesarian section. In Poland, the cesarian section rate is one of the highest in Europe, with the rate reaching approximately 48% in 2024 [31,32]. This figure significantly exceeds the World Health Organization’s recommended maximum rate of 15%, which underscores the need for improved strategies in prenatal care. The high rate of cesarian deliveries in Poland may be attributed to various factors, including medical and non-medical reasons, as well as patient and provider attitudes toward delivery methods. Interestingly, our findings, which suggest a potential benefit of physical activity on reducing cesarian sections, are consistent with results from other Polish studies that have explored similar relationships between maternal activity levels and birth outcomes [31]. Considering these findings, it is important to focus on educating women on the benefits of physical activity during pregnancy, not only for general health but also in relation to the mode of delivery. As cesarean section rates continue to rise, promoting an active lifestyle and offering tailored prenatal exercise programs may be essential in reducing unnecessary medical interventions and improving maternal and neonatal outcomes.

In our study, we found no significant association between maternal hemoglobin levels and physical activity during pregnancy. This lack of correlation is consistent with the findings of some other studies, which have also failed to identify a direct relationship between physical activity and hemoglobin levels in pregnant women [18]. While physical activity has well-established benefits for overall maternal health, including improved cardiovascular function and increased blood circulation, its impact on hemoglobin concentrations remains inconclusive. One potential explanation for this finding is that hemoglobin levels during pregnancy are influenced by a complex interplay of factors, including diet, iron supplementation, genetics, and the physiological changes associated with pregnancy itself. Pregnancy leads to an increase in blood plasma volume, which can dilute hemoglobin levels, especially in the second trimester, when plasma volume expansion is most pronounced. Additionally, iron deficiency is common during pregnancy and can have a more significant impact on hemoglobin levels than physical activity, which may not directly address iron status. Furthermore, as physical activity during pregnancy varies greatly in terms of type, intensity, and duration, it is possible that any potential effect on hemoglobin could be masked by other confounding variables, such as dietary intake, pre-existing anemia, or comorbidities. This could explain why we did not observe a statistically significant relationship between physical activity and hemoglobin levels in our study group. Interestingly, some studies have suggested that regular physical activity may improve iron absorption and utilization [18,33], but the effect might be subtle or overshadowed by other factors that more strongly influence maternal hemoglobin levels, such as iron intake and gastrointestinal absorption. Additionally, the impact of physical activity may vary depending on the individual’s baseline health status, with physically active women potentially having better overall health, which could mitigate the effect of physical activity on hemoglobin levels. Furthermore, the impact of physical activity on hemoglobin levels may also be influenced by the type of activity performed. In light of these considerations, future research focusing on the role of physical activity in maternal iron metabolism and hemoglobin levels should account for a broader range of influencing factors, including nutritional status, baseline hemoglobin levels, and the specific types of exercise performed. Longitudinal studies could provide a clearer picture of how physical activity influences hemoglobin levels across different stages of pregnancy.

Pregnant women in our study declared various types of physical activity undertaken during pregnancy, with walking being the most frequently chosen activity (n = 83). This is consistent with findings from DiPietro et al. [30], who identified the beneficial effects of aerobic exercise, including walking, on maternal health, particularly in reducing postpartum depression symptoms. Additionally, Ribeiro et al. [6], in their meta-analysis, showed that aerobic exercise during pregnancy positively influenced the reduction in postpartum weight retention. Walking, being a low-impact and accessible form of exercise, is often the preferred choice among pregnant women, as it is both easy to perform and associated with minimal risk. However, it is important to highlight that while walking offers significant health benefits, it is essential for pregnant women to incorporate a variety of physical activities into their routine to address different aspects of health, including cardiovascular fitness, muscle strength, and flexibility. Recent studies have emphasized the importance of not only aerobic exercise like walking, but also the inclusion of moderate-intensity activities such as resistance training and even high-intensity interval training (HIIT), which is becoming an increasingly popular option for pregnant women looking to maintain or improve fitness during pregnancy. Programs like HIIT Mamma [34] are designed to provide a structured and safe approach for pregnant women to engage in high-intensity exercise. Such programs, if implemented correctly, can contribute to better cardiovascular health, improved weight management, and a more efficient postpartum recovery.

Interestingly, our study also revealed a trend where the number of different types of physical activity undertaken decreased with each subsequent pregnancy. This finding may be attributed to the increased responsibilities associated with older children, which limit the time and energy available for physical activity. The observation aligns with the existing literature that highlights the impact of familial and caregiving duties on the physical activity levels of mothers [35,36]. Despite this, the study also noted that the level of physical activity was not influenced by the age of the pregnant woman. This suggests that other factors, such as personal preferences, time constraints, and lifestyle, may play a more significant role in determining the type and frequency of exercise during pregnancy.

In our study, we found that the total amount of physical activity did not have a significant influence on the child’s birth weight. However, an association was observed between the frequency of physical activity and birth weight. Specifically, as the frequency of physical activity increased, the number of normal-weight births (within the range of 2500–3999 g) also increased. Interestingly, in the subgroup of women who reported engaging in physical activity 5–7 times a week, there was an increase in the rate of macrosomia (birth weight ≥ 4000 g). In each group of physically active women, we observed a decrease in the number of births classified as small for gestational age (SGA). These findings are consistent with those of some studies, which report a lack of significant effect of physical activity on birth weight [37,38] while other research suggests a positive association [39]. This suggests that further studies with more specific inclusion criteria are needed to clarify the relationship between physical activity and birth weight. Some systematic review [40] indicate that the relationship between physical activity and birth weight may depend on factors such as the timing and duration of exercise during pregnancy. Studies have demonstrated that structured exercise programs initiated before the 15th week of pregnancy, particularly in overweight or obese women, may reduce the risk of macrosomia. However, other studies found no significant effect when exercise was introduced later or performed for a shorter duration. This could suggest that in our study, women who were more physically active may have had other contributing factors influencing the increased risk of macrosomia, such as dietary habits or other lifestyle aspects. This suggests that further studies with more specific inclusion criteria are needed to clarify the relationship between physical activity and birth weight. Additionally, in our study, the number of different physical activities undertaken by the participants did not significantly correlate with birth weight.

In our study, we examined the relationship between the intensity and frequency of physical activity during pregnancy and various neonatal outcomes, including the baby’s saturation after birth, APGAR scores, and cord blood pH. Our findings suggest a positive effect of the intensity and frequency of physical activity on the baby’s saturation after birth, indicating that more frequent or intense physical activity during pregnancy might improve neonatal oxygenation levels at birth. This observation aligns with those of some studies suggesting that maternal physical activity enhances placental function and, by extension, fetal oxygenation [41,42]. Enhanced maternal fitness and improved cardiovascular health could lead to better placental perfusion, resulting in a more favorable oxygen supply to the fetus, which may explain the increased oxygen saturation in the newborns.

However, despite the positive effects observed on neonatal oxygen saturation, our results indicated no significant influence of maternal physical activity on APGAR scores at 1 and 5 min post-delivery. These scores, which assess the newborn’s immediate postnatal condition, including heart rate, respiratory effort, muscle tone, reflex response, and color, were unaffected by the level of physical activity during pregnancy. This is consistent with previous research suggesting that while physical activity can improve overall maternal health, its direct effect on neonatal vitality, as reflected by the APGAR score, may be limited [43].

An interesting and somewhat unexpected finding in our study was the negative relationship between physical activity and cord blood pH levels. As physical activity increased, we observed a decrease in the pH levels of the cord blood. This result is contrary to the expected positive outcomes of physical activity, where better oxygenation and overall maternal health could lead to more optimal pH levels. A possible explanation could be that increased physical activity could lead to a more substantial mobilization of lactate during maternal exercise, potentially influencing the fetal acid–base balance. Additionally, the correlation between physical activity and pH levels may be confounded by other factors, such as the intensity of exercise, maternal health conditions, or intra-partum events, which were not fully accounted for in our study design. Our findings suggest that few researchers have compared maternal physical activity levels with umbilical cord blood pH, and the studies conducted in this area do not indicate a significant relationship between physical activity and umbilical cord blood pH values [43,44,45]. Overall, while our study supports the positive effect of physical activity on neonatal oxygenation, the lack of significant impact on APGAR scores and the unexpected association with decreased cord blood pH suggest that further investigation is needed. It is important to consider the complex interactions between exercise intensity, maternal metabolism, and fetal outcomes, as well as other potential confounders that may contribute to these findings. Future research with larger sample sizes, more detailed maternal health assessments, and the inclusion of other measures of fetal wellbeing may provide more comprehensive insights into the potential risks and benefits of physical activity during pregnancy.

### 4.1. Limitation

One of the limitations of the study was the relatively small sample size, the short data collection period and the subjective nature of the self-reporting tool, which could lead to reporting errors. Additionally, the study did not account for other factors influencing the course of pregnancy and the health of the mother and child, such as nutritional status or stress levels, which may have confounded the results. Although the study was prospective, it would benefit from more precise measurement tools, such as the Physical Activity in Pregnancy Questionnaire or accelerometers, to enhance the reliability of the results.

Another limitation of this study is the overrepresentation of individuals with higher education in our sample. This is likely due to the study being conducted in an area with several academic institutions, which attract individuals with higher educational backgrounds. Additionally, these institutions may contribute to internal migration, resulting in a higher proportion of highly educated individuals in the region. While this overrepresentation reflects broader trends in Poland [46,47,48,49], where the level of higher education, particularly among women, has been steadily increasing, it may limit the generalizability of our findings to populations with lower educational attainment. Future research should aim to include more diverse populations to better capture the variety of educational backgrounds across different regions.

### 4.2. Strengths

The strength of the study lies in its reliance on medical records, which provide an objective and reliable reflection of the mother and child’s health, as documented by healthcare professionals. This helps eliminate subjectivity in the assessment of key health indicators. Additionally, the data were collected from a single center in Poland, allowing for a more homogeneous study sample and minimizing the impact of ethnic variability on the results. While this may limit the generalizability of our findings, it also reduces potential confounding factors and allows for a more precise assessment of the studied associations. Future research on more diverse populations is needed to confirm these findings. 

## 5. Conclusions

The results of our study demonstrate an impact of physical activity on the health of both the mother and the newborn after childbirth. However, in many areas, further research is needed to establish clearer links. Importantly, the frequency of physical activity appears to be more beneficial than the intensity. Regular physical activity is essential not only in the preconception period but also during pregnancy, as it positively influences the well-being and self-perception of pregnant women. Although physical activity alone did not have a significant effect on the duration, mode of delivery, or birth weight of the newborn, it is important to note that it is just one of many factors contributing to these outcomes. Therefore, it is crucial to continue research in this area, especially in light of the global trend of insufficient adherence to minimum physical activity standards. Public health interventions should focus on promoting active and healthy lifestyles, which will benefit both current and future generations.

## Figures and Tables

**Table 1:** Characteristics of the study group.

Variable	Value	*n*	%
Place of residence	Village	34	16.50
	City up to 50,000 inhabitants	30	14.56
	City of 50,000 to 100,000 inhabitants	23	11.17
	City of 100,000 to 250,000 inhabitants	7	3.40
	City of 100,000 to 500,000 inhabitants	6	2.91
	City over 500,000 inhabitants	104	50.49
Education	Primary	5	2.43
	Basic vocational	14	6.80
	Secondary	44	21.36
	Higher	143	69.42
Marital status	Single	47	22.82
	Married	157	76.21
	Widowed	1	0.49
	Divorced	1	0.49
Economic status *	Average	36	17.48
	Good	169	82.04
Number of children	One child (nulliparous at the beginning of the study)	101	49.03
	Two children	52	25.24
	Three children	27	13.11
	Four and more children	26	12.62

* Good economic situation—characterized by financial stability, the ability to meet both basic and additional needs (e.g., saving, travel, investment), good housing conditions, and access to social capital. Individuals in this category do not experience financial difficulties. Average economic situation—indicates moderate financial stability, where basic needs are met, but access to higher-value goods (e.g., savings, travel, investments) may be limited. Budget management is necessary, though serious financial struggles are not present.

**Table 2:** Descriptive statistics of selected pregnancy-related variables.

Tested Variable	*M* ± *SD*
Age at delivery [years]	31.15 ± 4.92
Pre-pregnant BMI	24.48 ± 5.29
Gestational weight gain [kg]	13.42 ± 5.62
Birth weight [g]	3332.82 ± 602.08
Gestational age at birth (weeks)	38.80 ± 2.78
Newborn cord blood pH	7.27 ± 0.09
Maternal blood hemoglobin level	12.47 ± 1.40

**Table 3:** Analysis of perineal rupture in relation to physical activity during pregnancy.

Physical Activity During Pregnancy		Perineal Rupture							
		Lack of		1st Degree		2nd Degree		3rd Degree	
		*n*	*%*	*n*	*%*	*n*	*%*	*n*	*%*
Frequency (per week)	0	30	81.08	4	10.81	2	5.41	1	2.70
	1–2	36	69.23	4	7.69	12	23.08	0	0.00
	3–4	63	78.75	8	10.00	5	6.25	4	5.00
	5–7	21	56.76	6	16.22	9	24.32	1	2.70
Intensity	Low	82	70.09	14	11.97	17	14.53	4	3.42
	Moderate	65	77.38	8	9.52	9	10.71	2	2.38
	High	3	75.00	0	0.00	1	25.00	0	0.00
Duration	<20	43	82.69	5	9.62	3	5.77	1	1.92
	20–30	20	76.92	3	11.54	2	7.69	1	3.85
	30–60	30	63.83	5	10.64	9	19.15	3	6.38
	>60	57	70.37	9	11.11	14	17.28	1	1.23

**Table 4:** Frequency analysis of labor duration in relation to physical activity during pregnancy.

Physical Activity During Pregnancy	Labor Duration			
	I Stage		II Stage	
	*r_s_*	*p*	*r_s_*	*p*
Frequency	0.17	0.037	0.07	0.359
Intensity	0.21	0.012	0.08	0.252
Duration	0.13	0.129	0.18	0.012

*r_s_*—Spearman’s coefficient, *p*—significance.

**Table 5:** Frequency analysis of the mode of birth in relation to physical activity during pregnancy.

Physical Activity During Pregnancy		Mode of Birth			
		VB		C-Section	
		*n*	*%*	*n*	*%*
Frequency (per week)	0	21	56.76	16	43.24
	1–2	35	67.31	17	32.69
	3–4	54	67.50	26	32.50
	5–7	29	78.38	8	21.62
Intensity	Low	54	64.29	30	35.71
	Moderate	80	68.38	37	31.62
	High	4	100.00	0	0.00
Duration	<20	27	51.92	25	48.08
	20–30	34	72.34	13	27.66
	30–60	59	72.84	22	27.16
	>60	19	73.08	7	26.92

**Table 6:** Correlation analysis between maternal hemoglobin levels and physical activity during pregnancy.

Physical Activity During Pregnancy	Maternal Hemoglobin Levels	
	*r_s_*	*p*
Frequency	0.04	0.604
Intensity	0.07	0.356
Duration	0.02	0.730

*r_s_*—Spearman’s coefficient, *p*—significance.

**Table 7:** Correlation analysis between maternal age and physical activity during pregnancy.

Physical Activity During Pregnancy	Maternal Age	
	*r_s_*	*p*
Frequency	−0.11	0.123
Intensity	−0.10	0.141
Duration	−0.11	0.105

*r_s_*—Spearman’s coefficient, *p*—significance.

**Table 8:** Correlation analysis of birth weight and maternal physical activity during pregnancy.

Physical Activity During Pregnancy	Birth Weight	
	*r_s_*	*p*
Frequency	0.04	0.558
Intensity	−0.00	0.981
Duration	0.08	0.274

*r_s_*—Spearman’s coefficient, *p*—significance.

**Table 9:** Frequency analysis of birth weight relative to gestational age considering physical activity during pregnancy.

Physical Activity During Pregnancy		Birth Weight Relative to Gestational Age					
		LBW		Appropriate		Macrosomia	
		*n*	*%*	*n*	*%*	*n*	*%*
Frequency (per week)	0	7	18.92	26	70.27	4	10.81
	1–2	4	7.69	43	82.69	5	9.62
	3–4	4	5.00	73	91.25	3	3.75
	5–7	1	2.70	29	78.38	7	18.92
Intensity	Low	10	11.90	65	77.38	9	10.71
	Moderate	5	4.27	102	87.18	10	8.55
	High	1	25.00	3	75.00	0	0.00
Duration	<20	9	17.31	38	73.08	5	9.62
	20–30	3	6.38	40	85.11	4	8.51
	30–60	2	2.47	71	87.65	8	9.88
	>60	2	7.69	22	84.62	2	7.69

**Table 10:** Correlation analysis of neonatal parameters and physical activity during pregnancy.

Physical Activity During Pregnancy	Neonatal Parameters							
	Saturation		Apgar 1 min		Apgar 5 min		Cord Blood pH	
	*r_s_*	*p*	*r_s_*	*p*	*r_s_*	*p*	*r_s_*	*p*
Frequency	0.16	0.024	0.04	0.596	0.04	0.590	−0.17	0.018
Intensity	0.20	0.005	0.06	0.359	0.08	0.253	−0.11	0.126
Duration	0.05	0.520	−0.03	0.639	−0.01	0.885	−0.15	0.040

*r_s_*—Spearman’s coefficient, *p*—significance.

## Data Availability

The original contributions presented in the study are included in the article, further inquiries can be directed to the corresponding author.
